# GluN2B but Not GluN2A for Basal Dendritic Growth of Cortical Pyramidal Neurons

**DOI:** 10.3389/fnana.2020.571351

**Published:** 2020-11-13

**Authors:** Steffen Gonda, Jan Giesen, Alexander Sieberath, Fabian West, Raoul Buchholz, Oliver Klatt, Tim Ziebarth, Andrea Räk, Sabine Kleinhubbert, Christian Riedel, Michael Hollmann, Mohammad I. K. Hamad, Andreas Reiner, Petra Wahle

**Affiliations:** ^1^Developmental Neurobiology, Faculty of Biology and Biotechnology, Ruhr University Bochum, Bochum, Germany; ^2^Cellular Neurobiology, Faculty of Biology and Biotechnology, Ruhr University Bochum, Bochum, Germany; ^3^Biochemistry I – Receptor Biochemistry, Faculty of Chemistry and Biochemistry, Ruhr University Bochum, Bochum, Germany

**Keywords:** rodent neocortex, ampakine CX546, biolistic transfection, GluN1, inhibition

## Abstract

NMDA receptors are important players for neuronal differentiation. We previously reported that antagonizing NMDA receptors with APV blocked the growth-promoting effects evoked by the overexpression of specific calcium-permeable or flip-spliced AMPA receptor subunits and of type I transmembrane AMPA receptor regulatory proteins which both exclusively modify apical dendritic length and branching of cortical pyramidal neurons. These findings led us to characterize the role of GluN2B and GluN2A for dendritogenesis using organotypic cultures of rat visual cortex. Antagonizing GluN2B with ifenprodil and Ro25-6981 strongly impaired basal dendritic growth of supra- and infragranular pyramidal cells at DIV 5–10, but no longer at DIV 15–20. Growth recovered after washout, and protein blots revealed an increase of synaptic GluN2B-containing receptors as indicated by a enhanced phosphorylation of the tyrosine 1472 residue. Antagonizing GluN2A with TCN201 and NVP-AAM077 was ineffective at both ages. Dendrite growth of non-pyramidal interneurons was not altered. We attempted to overexpress GluN2A and GluN2B. However, although the constructs delivered currents in HEK cells, there were neither effects on dendrite morphology nor an enhanced sensitivity to NMDA. Further, co-expressing GluN1-1a and GluN2B did not alter dendritic growth. Visualization of overexpressed, tagged GluN2 proteins was successful after immunofluorescence for the tag which delivered rather weak staining in HEK cells as well as in neurons. This suggested that the level of overexpression is too weak to modify dendrite growth. In summary, endogenous GluN2B, but not GluN2A is important for pyramidal cell basal dendritic growth during an early postnatal time window.

## Introduction

Dendritic growth is a highly dynamic process (Wong and Ghosh, 2002). Elongation and outgrowth of side branches and retraction-remodeling occur within short time windows of hours and days as has been shown for tadpole tectal neurons (Cline, 2001). The synaptotropic hypothesis postulates that immature branches probe for potential afferent partners, and become stabilized when afferent presynapses form stable contacts (Vaughn, 1989; Cline and Haas, 2008). More meaningful synaptic activity for instance evoked by rearing in an enriched environment leads to an enhanced dendritic complexity (Volkmar and Greenough, 1972). Repetitive maze training enhances branching of distal apical, but not basal dendrites of layer IV and V pyramidal cells in visual cortex even of adult animals (Greenough et al., 1979), whereas deprivation of activity leads to a stunted growth. Among the underlying mediators are glutamate receptors at excitatory axodendritic synapses. Depolarization is translated into calcium currents via voltage-gated channels and NMDA receptors (NMDARs) (Konur and Ghosh, 2005), activation of G-protein signaling (Van Aelst and Cline, 2004), local protein synthesis, and the release of trophic factors acting in a paracrine or autocrine manner (McAllister, 2000; Wong and Ghosh, 2002; Wirth et al., 2003).

The three classes of ionotropic glutamate receptors, AMPA receptors (AMPARs), kainate receptors (KARs) and NMDARs, are richly expressed in developing and adult cortical neurons (Hadzic et al., 2017). All contribute to dendritic growth (Wong and Ghosh, 2002). For developing neocortical pyramidal neurons selected AMPAR subunits, most effectively as flip-spliced variants, are important as well as the KAR GluK2, and both mediate their effects with a contribution by NMDARs (Hamad et al., 2011, 2014; Jack et al., 2018). The importance of NMDARs has been shown in tadpole tectal neurons which fail to grow when inhibited with 100 μM of the non-selective NMDAR antagonist APV, whereas AMPARs and sodium channel activity were not yet important at these early stages (Rajan and Cline, 1998). Similarly, blocking NMDARs with CGP 40116 from postnatal day 1–21 in rats *in vivo* reduces the length of basal, but not apical dendrites of prefrontal cortical pyramidal cells (Wedzony et al., 2005). These studies relied on non-selective antagonists which do not allow to differentiate between GluN2B- and GluN2A-containing receptors; the subunits known to switch during development (Sheng et al., 1994; Traynelis et al., 2010). The recent generation of GluN2A-preferring antagonists now provide the tools to perform such a differentiation. Thus, we aimed to characterize in organotypic slice cultures the involvement of GluN2A and GluN2B for dendritic growth of cortical neurons.

## Materials and Methods

### Preparation of Organotypic Cultures (OTC)

Organotypic cortex cultures (OTCs) were prepared as described (Hamad et al., 2011; Jack et al., 2018) from pigmented Long-Evans rats from the in-house breeding facility with approval from the Ruhr-University Animal Research Board and the State of North Rhine-Westphalia. Briefly, visual cortex was explanted at postnatal day 0/P1 (P0, day of birth) and cut into 350 μm thick coronal slices using a McIlwain tissue chopper. Cultures were fed three times a week with semiartificial medium containing 25% adult horse serum, 25% Hank’s balanced Salt Solution, 50% Eagle’s Basal Medium, 1 mM L-glutamine (all from Life Technologies, Karlsruhe, Germany) and 0.65% D-Glucose (Merck, Darmstadt, Germany). To inhibit glial growth, 10 μl of an antimitotic cocktail consisting of uridine, cytosine-β-D-arabinofuranoside and 5-fluorodeoxyuridine (each stock 1 mM, all from Sigma; 10 μM final concentration for each) was applied at DIV 2 for 24 h.

### Plasmid Transfection

OTC were gene gun-transfected as described (Hamad et al., 2011; Jack et al., 2018). All plasmids (Table 1) were prepared as endotoxin-free solutions using the EndoFree Plasmid Maxi Kit (Qiagen, Cat No./ID: 12362). Plasmid stocks were diluted to 1 μg/μl and stored at −20°C. Briefly, cartridges were prepared by coating 7 mg gold microparticles (1 μm diameter; Bio-Rad) with 10 μg of the selected plasmids. Cultures were blasted (Helios Gene Gun, Bio-Rad, Munich, Germany) with 180 psi helium pressure.

**TABLE 1:** Plasmids and NMDA receptor antagonists.

**Plasmid, Promoter**	**Source**	**Catalog #**
pEGFP-N1 in pcDNA3.0, CMV	Clontech, Heidelberg, Germany	cat# 632370
pmCherry-N1, pcDNA3.0, CMV	Clontech, Heidelberg, Germany	cat# 632523
mKate (pTagFP635), CMV	Gift from Dr. Thomas Hughes	n.a.
GluN1-1a-pcDNA3, CMV	Gift from Michael Hollmann	n.a.
GluN2A-pcDNA3, CMV	Gift from Michael Hollmann	n.a.
GluN2A-pEGPF, CMV	Gift from Michael Hollmann	n.a.
GluN2B-pcDNA3, CMV	Gift from Michael Hollmann	n.a.
GluN2B-pEYPF, CMV	Gift from Michael Hollmann	n.a.
**Reagent, final concentration**		
Ifenprodil, 10 μM	Sigma-Aldrich	cat# I2892
Ro25-6981, 5 μM	Sigma-Aldrich	cat# R7150
NVP-AAM077, 200 nM	Sigma-Aldrich	cat# P1999
TCN201, 10 μM	Tocris	cat# 4154
CX546, 5 μM, 25 μM	Sigma-Aldrich	cat# C271
NMDA, 50 μM	Sigma-Aldrich	cat# M3262
DL-AP5, 5 μM	Alomone Labs	cat# D-140
L-glutamate, 500 μM and 1 mM	Sigma-Aldrich	cat# 49449

### Pharmacological Treatment

The first experiment was designed as follow-up of previous work (Hamad et al., 2011). We tested if an activation of AMPARs with a cognitive enhancer, the ampakine CX546, could alter dendritic growth. For the CX546 experiment, EGFP was transfected at DIV 4 and DIV 9 with analysis at DIV 10 and DIV 20, respectively. CX546 (Sigma) was dissolved in DMSO and applied at 5 μM or 25 μM final concentration to the medium; concentrations were kept low to avoid a knockdown of glutamate receptor expression reported to occur with high concentrations (Lauterborn et al., 2003). DMSO was used as vehicle control at a concentration not exceeding 0.1% in the medium. CX546 at 30 μM has been shown to alter firing pattern *in vitro* (Lu et al., 2016), to restore long-term potentiation at 25 μM in a Neto1 protein null mouse hippocampus by indirectly enhancing NMDAR synaptic currents (Ng et al., 2009), and to enhance neurite growth of cultured subventricular zone progenitor cells at 5–50 μM (Schitine et al., 2012).

For the NMDAR experiments, OTC were transfected with EGFP either at DIV 4 with analysis at DIV 10, or at DIV 14 with analysis at DIV 20. In the earlier time window GluN2 receptor antagonists were applied at DIV 7 and DIV 9, and in the later time window at DIV 15 and DIV 17, always combined with a medium change. For the recovery experiment, OTC were transfected at DIV 4, treated with ifenprodil at DIV 6 and DIV 8, switched to normal medium at DIV 10, and allowed to recover until DIV 15.

We decided to study in parallel two antagonists selective for GluN2B receptors, ifenprodil [dissolved in ethanol and further diluted with H_2_O] and Ro25-6981 [dissolved in DMSO], and two antagonists for GluN2A, NVP-AAM077 [dissolved in H_2_O] and TCN201 [dissolved in DMSO] (Table 1 for concentrations in the medium). Control cultures were mock-stimulated with DMSO at the final concentration or with H_2_O. Biophysically, ifenprodil and Ro25-6981 have the same mechanism of action at the N-terminal domain; and for ifenprodil a 10 μM concentration has been used for the 2-day incubation of the 150–300 μm thick slice cultures although 3 μM has been shown to already elicit the maximal inhibition (Gray et al., 2011). The two selected GluN2A antagonists differ in action with NVP-AAM077 being a competitive antagonist at the glutamate binding site and TCN201 acting at the glycine cofactor binding site (Neyton and Paoletti, 2006; Traynelis et al., 2010; Edman et al., 2012; Hansen et al., 2012, 2014; Tovar et al., 2013). Subunit-specific inhibition has been shown to deliver results that differ from those obtained with APV (He et al., 2013). The use of two antagonists for each subunit should reduce the probability of false negative or false positive results, and we expected the two pairs of antagonists to deliver comparable results, which turned out to be the case.

Finally, we followed the design of our previous studies overexpressing AMPAR and KAR subunits and their accessory proteins (Hamad et al., 2011, 2014; Jack et al., 2018). We attempted to express plasmids encoding the GluN2A and GluN2B subunit together with EGFP as reporter (for morphometry), or to express plasmids encoding EGFP-tagged GluN2A and EYFP-tagged GluN2B together with mCherry as reporter (for visualization). Further, we expressed GluN1-1a alone and GluN1-1a together with GluN2B (for morphometry). Transfection was done at DIV 5 followed by staining and analysis at DIV 10.

### Immunostaining

OTCs were fixed with 37°C 4% paraformaldehyde in 0.1 M phosphate buffer pH 7.4 for 2 h, rinsed, permeabilized for 30 min with 0.3% Triton X-100 in phosphate buffer, blocked with TBS/BSA solution, and incubated overnight in mouse anti-GFP antibody (1:1000; clone GSN24, Sigma-Aldrich, RRID: AB_563117). To visualize the neuronal morphology via the reporter protein, staining proceeded with biotinylated goat anti-mouse (1:1000; Dako A/S, Glostrup, Denmark, product no. E043301-2) for 3 h, followed by ABC reagent for 2 h (Vector Laboratories Inc., Burlingame, CA, United States, cat# PK-7100; RRID: AB_2336827), a HRP reaction with 3,3’-diaminobenzidine (DAB, Sigma-Aldrich, Steinheim, Germany) and H_2_O_2_. The resulting DAB product was intensified with 1% OsO_4_ (Sigma-Aldrich, Steinheim, Germany). Cultures were dehydrated and coverslipped in DEPEX (Sigma-Aldrich, Steinheim, Germany).

To visualize the overexpression, GluN2A-EGFP and GluN2B-EYFP tagged constructs were transfected together with mCherry fluorescence as reporter, and were stained with the mouse anti-GFP antibody developed with an Alexa 488-conjugated secondary (1:500; Thermo Fisher Scientific, cat# A-11001, RRID: AB_2534069) followed by embedding in ImmuMount or RIMS buffer.

### NMDA-Induced Dendritic Injury Assay

OTC were challenged with 50 μM NMDA in the medium under live imaging followed by fixation and staining for EGFP as described above. Antagonists were preincubated to allow diffusion into the slice cultures. The time of exposure is indicated in the figure legends. The percentage of transfectants displaying dendritic beading was determined after DAB immunostaining.

### Protein Blots

Lysates and blots were performed as described (Engelhardt et al., 2018; Neumann et al., 2019) with OTC from 3 preparations that were exposed to ifenprodil, and allowed to recover from ifenprodil treatment for 12h and 24h. Using membrane strips placed over the molecular weight range of the target protein of interest several proteins of distinct kDa can be assessed per lysate. Antibodies against the following proteins and epitopes were used: GAD-65/67 (mouse; clone 9A6, cat# #ADI-MSA-225-E, 1:2000; Enzo Life Science, Lörrach, Germany), phY1472 GluN2B (rabbit; 1:1000; Rockland Inc., cat# 612-401-C89; via Biomol, Hamburg, Germany), GluN2B (mouse; 1:1000; cat# 06-600, Merck Millipore, Darmstadt, Germany), GluN1 (mouse; 1:1000; NeuroMab, UC Davis, Davis, CA, United States), β-tubulin (mouse; 1:1000; cat# T5293, Sigma-Aldrich, Deisenhofen, Germany), β-actin (mouse; 1:4000; cat# 1978, Sigma-Aldrich, Deisenhofen, Germany), PSD-95 (rabbit; 1:1000; cat# 124 002, Synaptic Systems, Göttingen, Germany). Visualization was done with alkaline phosphatase-conjugated secondaries and reaction with 5-bromo-4-chloro-3-indolyl-phosphate/nitro blue tetrazolium (Promega, Mannheim, Germany). Blots were scanned, band intensities assessed with ImageJ followed by normalization to a housekeeping protein. Representative bands were presented together with the actin and tubulin bands.

### Expression, Patch-Clamp Recordings, and Immunocytochemistry of NMDA Receptor Constructs in HEK293T Cells

Functional expression of GluN2A-EGFP and GluN2B-EYFP was verified in whole-cell patch clamp recordings. Human embryonic kidney (HEK) 293T cells were grown in DMEM with 8% FBS at 37°C and 5% CO_2_ on plastic coverslips. Plasmid triple transfections were carried out using polyethylenimine 25.000 with ∼0.2 μg GluN1-1a and ∼0.1 μg GluN2A or GluN2B per ml medium; 0.1 μg mKate was added as transfection marker. To reduce detrimental effects due to NMDA receptor expression the medium was supplemented with 5 mM Mg^2+^ or 5 μM DL-AP5 (Alomone Labs, D-140). All other chemicals were from Sigma. Whole-cell recordings were performed 24–48 h after transfection on an inverse microscope (DMi8, Leica) using an Axopatch 200B patch-clamp amplifier, a Digidata 1550 A/D converter and pClamp 10.7 software (all Molecular Devices) and a micro-manipulator (Patchstar, Scientifica). In brief, patch pipettes (3–4 MΩ resistance) were pulled from borosilicate glass and filled with internal solution containing 122 mM CsCl, 2 mM NaCl, 2 mM MgCl_2_, 10 mM EGTA and 10 mM HEPES, pH 7.2. The coverslips were placed in external solution containing 138 mM NaCl, 1.5 mM KCl, 2.5 mM CaCl_2_, 50 μM glycine and 10 mM HEPES, pH 7.3. External solution and L-glutamate (500 μM or 1 mM; Sigma, cat# 49449) in external solution were applied by means of a gravity-driven perfusion system. Recordings were performed at 22–25°C in voltage-clamp mode at a holding potential of −70 mV. Experiments were repeated minimum three times after independent transfections and baseline corrected. For the evaluation of peak and steady-state currents only recordings with leak currents <500 pA and current changes >20 pA were taken into account.

For immunocytochemistry in HEK293T cells, the expression was performed as described above, but without co-transfection of mKate. After 24–48 h, the transfected HEK293T cells were washed with DPBS and fixed with 4% PFA for 15 min. After washing the cells with DPBS, unspecific binding sites were blocked with Roti-Immunoblock (Roth) and cells permeabilized with 0.25% Triton X-100 for 45 min. Cells were washed with DPBS and incubated with mouse anti-GFP antibody (Sigma, 1:1000) overnight at 4°C. Following multiple washing steps, the cells were incubated with the secondary antibody (Alexa Fluor 568, Invitrogen, cat# A10037) for 60 min at room temperature. Finally, the coverslips were washed and mounted with Roti-Mount FluorCare DAPI. Confocal imaging was performed on a Leica SP5 using 405, 488, and 561 nm laser lines. Fluorescence overlays were prepared using ImageJ/Fiji 1.53c.

### Morphological Analysis

For morphometrical analysis, immunostained neurons were reconstructed with the Neurolucida system (MicroBrightField, Inc., Williston, VT, United States) by trained observers blinded to conditions. All reconstructions were crosschecked by an observer blinded to condition for correctness and to classify the cell type. Pyramidal cells and multipolar sparsely spinous interneurons were classified by criteria of dendritic and axonal patterns. Pyramidal neurons were grouped as follows: those of layers II/III have an apical dendrite reaching into layer I, and for layers V/VI we consider those which have an apical dendrite ending in middle layers; thick and thin tufted large layer V pyramidal cells occurred too rarely and were not sampled (Hamad et al., 2014; Jack et al., 2018). Using a ZEISS Axioskop equipped with a discussion bridge we assessed with two trained observers blinded to conditions all completely stained neuronal transfectants per slice culture for symptoms of dendritic injury. We considered only completely labeled neurons with recognizable axons, because dendritic injury often starts with blebbing at distal dendrites. Data are presented as Tables and Sholl plots. Statistical analyses were done with Sigma Plot 12.3 (Systat Software, Erkrath, Germany). Non-parametric ANOVA on ranks tests with corrections for multiple testing (Dunn’s test) and/or non-parametric Mann–Whitney rank sum tests were conducted.

## Results

### No Effect of the Ampakine CX546 but Underdevelopment With Ifenprodil

The first experiment followed our earlier observation which showed that calcium-permeable and/or flip splice variants (with longer channel open times) of specific GluA subunits increase complexity of pyramidal cell apical, but not basal dendrites (Hamad et al., 2011). We tested if a cognitive enhancer can alter dendritic growth. The classical type 1 ampakine CX546 slows desensitization and deactivation of AMPARs, prolongs the synaptic response, and strengthens long-term potentiation (Arai and Kessler, 2007). This can increase the synthesis of BDNF which stays at somewhat higher levels for several hours and activates TrkB receptors in spines (Lauterborn et al., 2000, 2003, 2009; Montgomery et al., 2009). BDNF via TrkB promotes dendritogenesis (McAllister, 2000; Wirth et al., 2003), and chronic ampakine stimulation has been reported to prevent age-dependent dendritic pruning (Lauterborn et al., 2016). Since previous data had indicated a role of NMDARs (Hamad et al., 2011), we included a treatment with ifenprodil. However, the ampakine treatment at 5 μM from DIV 5 to DIV 10 did not alter dendritic complexity of pyramidal neurons (layers II/III tested) (Supplementary Table 1A). Further, acute wash-in of CX546 at concentrations up to 1 mM did not evoke dendritic beading in 10 DIV OTC, and there was no neuroprotection by ifenprodil (CX546: 4.3 + −1.1% neurons with dendritic beading, 227 neurons from 5 OTC; control, vehicle stimulated: 3.8 + −1.0% neurons with dendritic beading, 233 neurons from 5 OTC; ifenprodil pretreatment followed by ampakine: 3.9 + −1.3% neurons with dendritic beading, 289 neurons from 4 OTC; this is in the <10% range typical for untreated OTC; Hamad et al., 2011). The inefficiency might be due to the low concentration of endogenous AMPARs at DIV 5–10. However, a daily stimulation with CX546 at a higher concentration (25 μM final concentration in the medium, medium change every second day) in more mature cultures from DIV 10–20 also did not alter dendritic complexity of pyramidal neurons of supra- and infragranular layers (Supplementary Table 2A). Further, multipolar non-pyramidal neurons with smooth or sparsely spinous dendrites were neither altered by CX546 exposure at DIV 10 (Supplementary Table 1B) nor at DIV 20 (Supplementary Table 2B). The result is in line with the interpretation of previous studies. Potentiating AMPARs for instance by overexpressing the modulatory type II TARPs does not alter dendritic complexity whereas increasing the number of plasma membrane receptors does promote dendritic growth (Hamad et al., 2011, 2014). The unexpected result was that pyramidal neurons reconstructed from the DIV 10 ifenprodil-treated cultures showed significantly shorter and less branched basal dendrites (Supplementary Table 1A). Interneurons, however, were not affected (Supplementary Table 1B). This led us to study the role of endogenous GluN2B-containing receptors for pyramidal cell dendritic growth.

### Blocking of GluN2B but Not GluN2A Prevents NMDA-Induced Dendritic Injury

**FIGURE 1:**
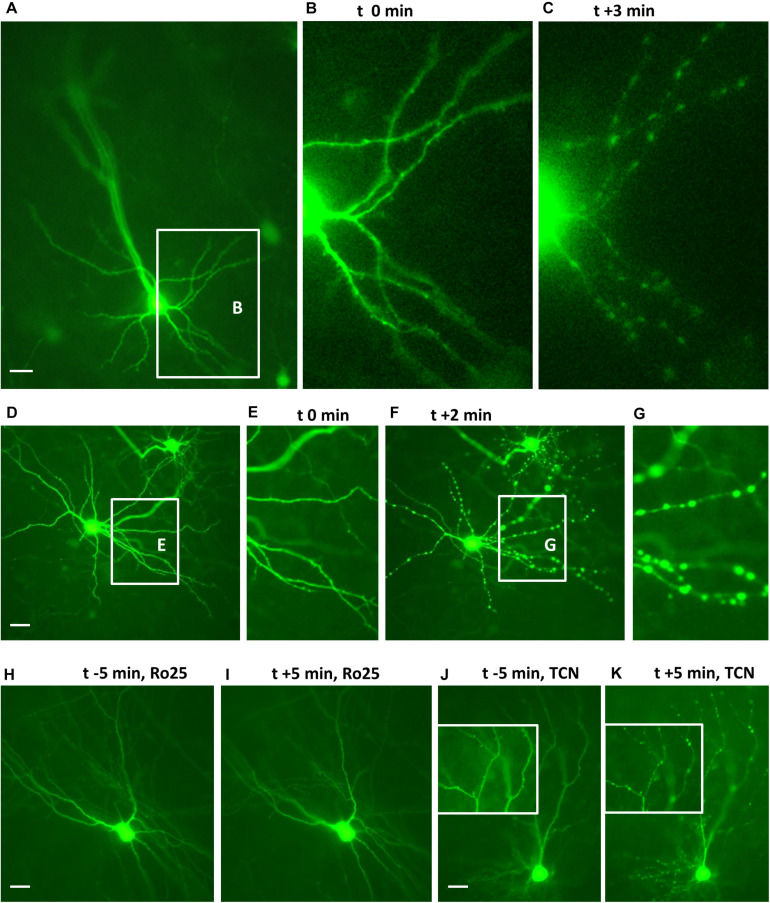
Endogenous GluN2 receptors are functional on DIV 10 neurons. Cultures with live EGFP-expressing neurons were challenged with 50 μM NMDA to evoke dendritic beading. **(A–C)** Pyramidal neuron before and 3 min (t + 3 min) after application of NMDA. The inset in **(A)** shows basal dendrites which are seen at higher magnification in **(B,C)**. **(D–G)**: Interneuron before **(D,E)** and 2 min after NMDA application **(F,G)**. The insets in **(D,F)** show the dendrites which are at higher magnification in **(E,G)**, respectively. **(H,I)**: Interneuron protected from the NMDA-induced dendritic beading by preincubation with 5 μM Ro25-6981. **(J,K)** Pyramidal cell not protected from the NMDA-induced dendritic beading by preincubation with 10 μM TCN201; insets show details of apical dendritic branches at higher magnification before and 5 min after application of NMDA. Pial surface is to the upper left for **(A–I)** and to the top for **(J,K)**, Scales: 10 μm.

We have previously reported that GluN1, GluN2A, and GluN2B are expressed in OTC at DIV 5, DIV 10, and DIV 15–20 and the expression level of the three proteins increases with age (Engelhardt et al., 2018; Neumann et al., 2019). First, we confirmed that young neurons in OTC have functional NMDARs on the cell surface. GFP-transfected OTC were challenged at DIV 10 with 50 μM NMDA to evoke dendritic beading as previously shown (Hamad et al., 2011; Jack et al., 2018). Images of live neurons were taken at 5 min before, or just before (0 min) adding NMDA and subsequently every minute for up to 10 to up to 15 min. This revealed that both, pyramidal neurons (Figures 1A–C) and multipolar sparsely spinous interneurons (Figures 1D–G) responded with dendritic beading to NMDA. Preincubation for 1 h with the GluN2B antagonist Ro25-6981 prevented dendritic beading as shown exemplarily for a multipolar neuron (Figures 1H,I). Preincubation for 1 h with the GluN2A antagonist TCN201 could not prevent dendritic beading as shown exemplarily for a pyramidal neuron (Figures 1J,K).

**FIGURE 2:**
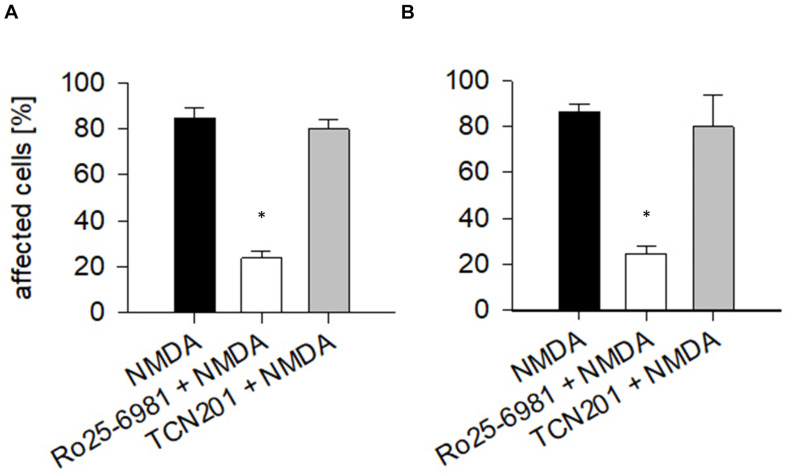
Endogenous NMDA receptors render neurons sensitive to NMDA. At DIV 10, the vast majority of **(A)** pyramidal cells and of **(B)** interneurons responded with dendritic beading to NMDA (50 μM) applied for 10 min. Preincubation with Ro25-6981 (5 μM) was neuroprotective (ANOVA on ranks versus control, *p < 0.05). TCN201 [10 μM] was unable to protect. Antagonists had been applied 60 min before the treatment with 50 μM NMDA, followed by immediate fixation. The average percentage of pyramidal neurons (in total 523 cells analyzed) and multipolar non-pyramidal neurons (in total 218 cells analyzed) with dendritic beading per culture was plotted as mean ± S.E.M. 6–10 OTC per condition were analyzed. Only completely stained neurons identifiable by axonal projections were assessed.

To quantify the effects, the proportion of EGFP-labeled neurons with dendritic beading was determined at DIV 10 in OTC that were exposed to 50 μM NMDA for 10 min followed by fixation and staining. About 80% of the transfected pyramidal neurons (Figure 2A) and multipolar interneurons (Figure 2B) displayed swollen varicosities along apical and basal dendrites after exposure to NMDA. Pretreatment with Ro25-6981 substantially prevented dendritic beading; in contrast, preincubation with TCN201 still resulted in over 80% affected neurons of both neuron classes (Figures 2A,B). Together, these results confirm the presence of functional GluN2B-containing NMDA receptors during the early time window.

### Blocking GluN2B Receptors Impairs Dendritic Growth of Young Pyramidal Neurons

Next, we quantified dendritic length and branching of pyramidal neurons of layers II/III and V/VI. All neurons had spiny dendrites and a primary axon descending toward deeper layers and white matter, giving rise to a few long-running, obliquely ascending collaterals. Since the two pairs of antagonists delivered comparable results (reported for supra- and infragranular pyramidal neurons at DIV 10 in Supplementary Table 3), we pooled the data sets for the two GluN2B antagonists (ifenprodil/Ro25-6981) and the two data sets for the GluN2A antagonists (NVP-AAM077/TCN201). The numbers of neurons and independent preparations (number of batches) are given in the Tables. Comparisons were done to the batch-internal controls which were also pooled.

**FIGURE 3:**
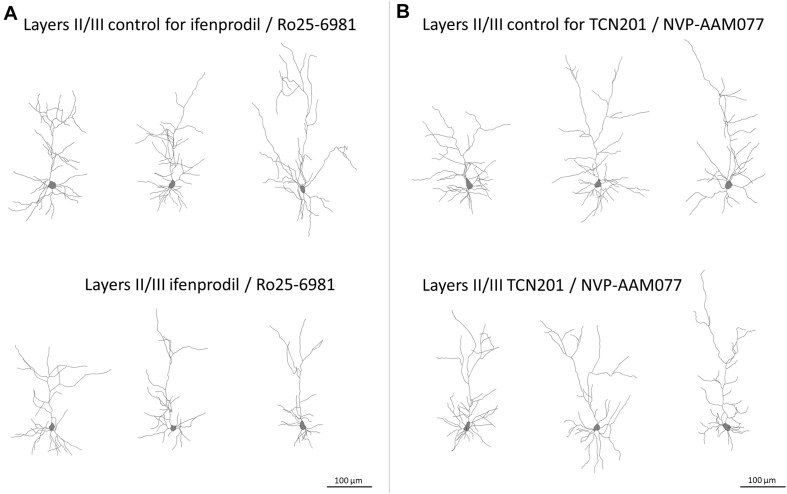
Skeletal drawings of representative pyramidal neurons at DIV 10. **(A)** Control and ifenprodil/Ro25-6981 treated pyramidal neurons of layers II/III. Note reduced dimensions of basal dendrites in the ifenprodil/Ro25-6981 treated neurons. **(B)** Control and TCN201/NVP-AAM077 treated pyramidal neurons of layers II/III, Scale = 100 μm.

The GluN2B subunit strongly influenced the formation of the basal dendritic tree of pyramidal cells in layers II/III and V/VI. Both, mean basal dendritic length and mean number of basal dendritic segments were significantly reduced after treatment with ifenprodil/Ro25-6981 (Figure 3 and Table 2A). However, the number of basal dendrites per neuron was not different between the groups (pyramidal cells of layers II/III: 4.07 ± 0.12 for EGFP control; 4.10 ± 0.16 for ifenprodil/Ro25-6981; and 4.17 ± 0.16 for NVP-AAM077/TCN201, ANOVA on ranks *p* = 0.76; pyramidal cells of layers V/VI: 3.87 ± 0.14 for EGFP control; 4.04 ± 0.15 for ifenprodil/Ro25-6981; and 3.5 ± 0.11 for NVP-AAM077/TCN201, ANOVA on ranks *p* = 0.074). This ruled out that the reduction of average basal dendritic length was caused by an increased sprouting of shorter processes. In the case of supragranular pyramidal cells the reduction of apical dendritic length and branching was only borderline (Table 2A). GluN2A seemed not to be involved in dendritic growth during the early time window since neither NVP-AAM077 nor TCN201 treatment changed the dendritic morphology. Both GluN2 subunits are expressed in cortical non-pyramidal interneurons (Hadzic et al., 2017). However, their dendritic growth seemed independent of endogenously expressed GluN2A- and GluN2B-containing NMDAR since none of the antagonists was able to alter the dendritic growth pattern (Table 2B).

**TABLE 2:** Effects of the GluN2B and GluN2A antagonists at DIV 10.

**(A) Pyramidal cells treated with ifenprodil/Ro25-6981 and TCN201/NVP-AAM077 from DIV 7–10**				
	**Pyramidal cells of layers II/III**		**Pyramidal cells of layers V/VI**	
**Condition (no. of batches)**	**ADL (*n*) Segments**	**BDL Segments**	**ADL (*n*) Segments**	**BDL Segments**
Control	1167 ± 50 (89) 32 ± 1.5	244 ± 13 7.0 ± 0.4	1016 ± 37 (75) 23 ± 1.2	272 ± 14 6.8 ± 0.3
Ifenprodil/Ro25-6981 (3 each)	1041 ± 40 (49/38) 28 ± 1.2	**194 ± 8.1 5.8 ± 0.3**	1000 ± 43 (37/40) 22 ± 1.1	**207 ± 10 5.5 ± 0.3**
*Mann–Whitney test vs. control*	*P = 0.1 P = 0.06*	*P = 0.014 P = 0.026*	*P = 0.41 P = 0.58*	*P < 0.001 P = 0.004*

Control	1193 ± 48 (101) 32 ± 1.3	237 ± 11 6.9 ± 0.3	1014 ± 39 (64) 22 ± 1.2	269 ± 14 7.1 ± 0.4
TCN201/NVP-AAM077 (2 and 3)	1121 ± 40 (25/60) 31 ± 1.3	229 ± 9 7.0 ± 0.3	1056 ± 42 (18/49) 24 ± 1.1	253 ± 14 6.6 ± 0.4
*Mann–Whitney test vs. control*	*P = 0.56 P = 0.86*	*P = 0.95 P = 0.6*	*P = 0.51 P = 0.15*	*P = 0.36 P = 0.3*

**(B) Interneurons treated with ifenprodil/Ro25-6981 and TCN201/NVP-AAM077 from DIV 7–10**				
**Condition (no. of batches)**	**MDL (n)**		**MDS**	**No. of PD**

Control	387 ± 17 (111)		7.0 ± 0.3	4.5 ± 0.1
Ifenprodil/Ro25-6981 (3 each)	352 ± 16 (56/37)		7.1 ± 0.4	4.6 ± 0.2
*Mann–Whitney test vs. control*	*P = 0.14*		*P = 0.5*	*P = 0.6*

Control	401 ± 26 (69)		6.4 ± 0.4	4.7 ± 0.2
TCN201/NVP-AAM077 (2 and 3)	395 ± 21 (18/58)		7.3 ± 0.4	4.6 ± 0.2
*Mann–Whitney test vs. control*	*P = 0.78*		*P = 0.1*	*P = 0.83*

*Antagonists were applied at DIV 7 and DIV 9. Given is the mean ± S.E.M, and, in italics, the *p*-values of the Mann–Whitney rank sum test. For pyramidal cells in **(A)**: ADL, apical dendritic length [μm]; BDL, the average basal dendritic length per cell [μm]; the number of dendritic segments; for interneurons in **(B)**: MDL, mean dendritic length [μm] per cell; MDS, mean dendritic segments; no. of PD, number of primary dendrites; *n*, number of neurons analyzed. In bold, the parameters that differ significantly from control.*

**FIGURE 4:**
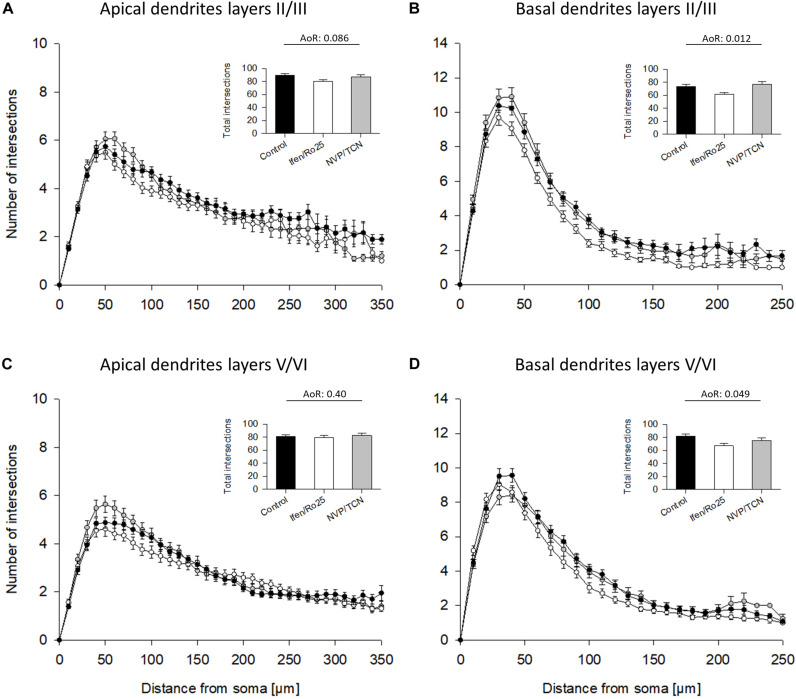
Sholl analyses of apical and basal dendrites of DIV 10 pyramidal cells of layers II/III and V/VI. **(A)** Sholl analyses of apical dendrites of pyramidal cells of layers II/III. Total intersections (mean with S.E.M.) are given in the insets. **(B)** Sholl analyses of basal dendrites of pyramidal cells of layers II/III showed a decrease of basal dendritic complexity in the ifenprodil/Ro25-6981 (Ifen/Ro25) treated neurons, but not NVP-AAM077/TCN201 (NVP/TCN) treated neurons between 50 and 200 μm from the soma. **(C)** Sholl analyses of apical dendrites of pyramidal cells of layers V/VI. **(D)** Sholl analyses of basal dendrites of pyramidal cells of layers V/VI showed a decrease of basal dendritic complexity in ifenprodil/Ro-6981 treated neurons, but not NVP-AAM077/TCN201 treated neurons between 50 and 150 μm distance from the soma. **(B,D)** ANOVA on ranks (AoR) versus control identified the significant reduction of total intersections in the ifenprodil/Ro25-6981 groups. The number of neurons analyzed per condition is given in Table 2.

The Sholl analyses confirmed the hypomorphy of ifenprodil/Ro25-6981 treated neurons (Figure 4). Apical dendrites of supragranular, but not infragranular pyramidal neurons seemed to have somewhat less branches (Figures 4A,B) although total intersections were not significantly different according to the ANOVA on ranks; further, apical dendrites were not different in the pilot experiment (Supplementary Table 1). The basal dendritic branching of supra- infragranular pyramidal neurons (Figures 4C,D) clearly remained below control within 50 to up to 200 μm distance from the soma suggesting a substantial reduction in dendritic branching. In contrast, the NVP-AAM077/TCN201 curves overlap with the control (Figures 4C,D).

### The Contribution of GluN2B-Containing Receptors to Dendritogenesis Was Temporally Limited

**FIGURE 5:**
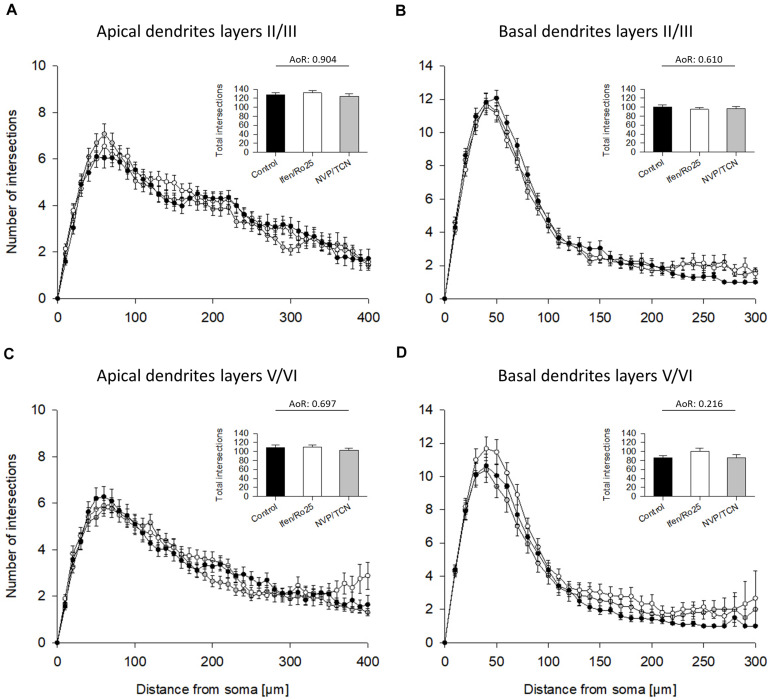
Sholl analyses of apical and basal dendrites of DIV 20 pyramidal cells of layers II/III and V/VI. **(A)** Sholl analyses of apical dendrites of pyramidal cells of layers II/III treated with ifenprodil/Ro25-6981 (Ifen/Ro25) and NVP-AAM077/TCN201 (NVP/TCN). Total intersections (mean with S.E.M.) are given in the insets. **(B)** Sholl analyses of basal dendrites of pyramidal cells of layers II/III. **(C)** Sholl analyses of apical dendrites of pyramidal cells of layers V/VI. **(D)** Sholl analyses of basal dendrites of pyramidal cells of layers V/VI. Note that the curves overlap with no obvious differences. ANOVA on ranks (AoR) run for the total intersections of the treatment groups versus control revealed no statistical differences. The number of neurons analyzed per condition is given in Table 3.

The GluN2 receptor composition switches during development from mainly GluN2B- to mainly GluN2A-containing heterodimeric and heterotrimeric receptors (Traynelis et al., 2010) with GluN1/2A/2B triheteromers presumably representing a majority of synaptic NMDAR in juvenile and adult neocortex and hippocampus (Sheng et al., 1994; Tovar et al., 2013; Stroebel et al., 2018). The switch depends on the maturational state of the neurons and also occurs in *in vitro* systems (Williams et al., 1993). Therefore, we tested a blockade of GluN2A and GluN2B receptors in more differentiated neurons at DIV 15–20. Again, results of the two antagonists of each pair went into the same direction, and we therefore pooled the DIV 20 data. None of the antagonists was able to significantly alter dendritic dimensions of pyramidal cells (Table 3A) or of interneurons (Table 3B). The Sholl analysis revealed a larger scatter at the higher age group, but by and large curves overlapped the control curve and total intersections were not different from control (Figure 5). This suggested that GluN2B has lost its role as dendritic modifier by DIV 15, and that GluN2A has no measurable role in dendritic growth.

**TABLE 3:** Effects of the GluN2B and GluN2A antagonists at DIV 20.

**(A) Pyramidal cells treated with ifenprodil/Ro25-6981 and TCN201/NVP-AAM077 from DIV 15–20**				
	**Pyramidal cells of layers II/III**		**Pyramidal cells of layers V/VI**	
**Condition (no. of batches)**	**ADL (*n*) Segments**	**BDL Segments**	**ADL (*n*) Segments**	**BDL Segments**
Control	1640 ± 72 (52) 32 ± 1.7	320 ± 19 7.5 ± 0.4	1387 ± 76 (45) 26 ± 1.4	273 ± 17 5.9 ± 0.4
Ifenprodil/Ro25-6981 (2 each)	1696 ± 72 (35/37) 31 ± 1.3	300 ± 14 6.9 ± 0.3	1397 ± 58 (26/32) 25 ± 1.3	307 ± 18 6.5 ± 0.4
TCN201/NVP-AAM077 (2 each)	1598 ± 58 (26/32) 32 ± 1.4	314 ± 20 7.6 ± 0.5	1272 ± 47 (33/14) 23 ± 1.3	276 ± 17 5.9 ± 0.4
*ANOVA on ranks vs. control*	*P = 0.82 P = 0.84*	*P = 0.82 P = 0.55*	*P = 0.36 P = 0.23*	*P = 0.40 P = 0.34*

**(B) Interneurons treated with ifenprodil/Ro25-6981 and TCN201/NVP-AAM077 from DIV 15–20**				
**Condition (no. of batches)**	**MDL (*n*)**	**MDS**		**No. of PD**

Control	524 ± 29 (61)	6.6 ± 0.4		4.6 ± 0.2
Ifenprodil/Ro25-6981 (2 each)	503 ± 30 (28/43)	6.7 ± 0.3		4.4 ± 0.2
TCN201/NVP-AAM077 (2 each)	452 ± 24 (37/35)	6.2 ± 0.3		4.6 ± 0.2
*ANOVA on ranks vs. control*	*P = 0.13*	*P = 0.38*		*P = 0.63*

*Antagonists were applied at DIV 15 and DIV 17. Given is the mean ± S.E.M, and, in italics, the *p*-values of an ANOVA on ranks. For pyramidal cells in **(A)**: ADL, apical dendritic length [μm]; BDL, the average basal dendritic length per cell [μm]; the number of dendritic segments; for interneurons in **(B)**: MDL, mean dendritic length [μm] per cell; MDS, mean dendritic segments; no. of PD, number of primary dendrites; *n*, number of neurons analyzed.*

### Basal Dendritic Growth Recovered After Ifenprodil Washout

**FIGURE 6:**
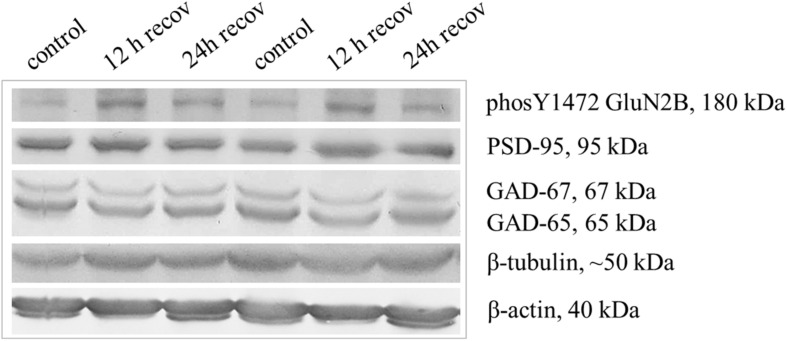
Higher expression of Y1472 phosphorylated GluN2B during recovery from ifenprodil treatment. The five nitrocellulose strips used to detect six proteins of different molecular weight [in kDa] derive all from one gel. Per lane, the lysate of 1 OTC (each about 1 mg wet weight) was loaded. Shown are arbitrarily paired OTC from one batch of cultures, two OTC at 12 h and two OTC at 24 h after washout of ifenprodil, and two OTC that were mock-stimulated, and harvested at the 24 h time point. Note that the level of GluN2B Y1472 phosphorylation was higher in the recovery cultures, the GAD-65 level was lower under ifenprodil and still lower after 12 h recovery; the PSD-95 levels were not affected. Tubulin and actin served as house-keeping proteins. For quantification see Table 5.

To test if neurons are able to recover from an early antagonism of GluN2B we transfected at DIV 4, and applied ifenprodil at DIV 6 and DIV 8. Ifenprodil can be washed out, and at DIV 10 we switched the OCT to medium without antagonist until fixation, staining and reconstruction at DIV 15. Basal dendritic complexity was at the level of the mock-stimulated control cells with apical dendrites (Table 4A) and dendrites of interneurons (Table 4B) unaltered. Protein blots (Figure 6) revealed that the ifenprodil treatment caused a reduction of GAD-65/67 expression which was still lower at 12 h recovery, but had returned to control values at 24 h after washout (Table 5). Developmental GAD-65/67 expression is known to be activity-dependent with a contribution of NMDA receptors (Qin et al., 1994; Patz et al., 2003). Interestingly, although total GluN2B protein was not altered under ifenprodil and after 24 h recovery (Table 5), the phosphorylation at tyrosine 1472 was increased after 12 and 24 h recovery (Figure 6 and Table 5). The phosphorylation serves to anchor GluN2B-containing receptors at the synapse and prevent internalization (Sanz-Clemente et al., 2013). GluN1 was not altered at 12 and 24 h recovery (Table 5). PSD-95, a marker for glutamatergic synapses, were neither altered under ifenprodil nor at during recovery (Figure 6 and Table 5). This suggested that pyramidal neurons within this time window can recover basal dendritic growth.

**TABLE 4:** Recovery of basal dendritic growth.

**(A) Pyramidal cells treated with ifenprodil from DIV 7–10 followed by recovery until DIV 15**				
	**Pyramidal cells of layers II/III**		**Pyramidal cells of layers V/VI**	
**Condition (no. of batches)**	**ADL (*n*) Segments**	**BDL Segments**	**ADL (*n*) Segments**	**BDL Segments**
Control	1526 ± 92 (36) 28 ± 1.9	308 ± 20 6.2 ± 0.5	1394 ± 80 (26) 26 ± 1.4	345 ± 32 6.9 ± 0.6
Ifenprodil (2)	1601 ± 106 (25) 29 ± 2.1	336 ± 31 7.3 ± 0.8	1303 ± 116 (20) 25 ± 1.9	284 ± 23 5.7 ± 0.5
*Mann–Whitney test vs. control*	*P = 0.49 P = 0.63*	*P = 0.73 P = 0.1*	*P = 0.35 P = 0.62*	*P = 0.32 P = 0.2*

**(B) Interneurons treated with ifenprodil from DIV 7–10 followed by recovery until DIV 15**				
**Condition (no. of batches)**	**MDL (*n*)**	**MDS**		**no. of PD**

Control	549 ± 45 (26)	7.2 ± 0.6		5.7 ± 0.3
Ifenprodil (2)	525 ± 45 (24)	9.3 ± 0.8		4.7 ± 0.4
*Mann–Whitney test vs. control*	*P = 0.68*	*P = 0.11*		*P = 0.02*

*Ifenprodil was applied at DIV 6 and DIV 8, and at DIV 10 OTC were switched to medium without antagonist until analysis at DIV 15. Length and branching of dendrites of pyramidal cells **(A)** and of interneurons **(B)** were no longer different from control cells. The weak significance for the number of primary dendrites is presumably due to sampling; primary dendrites had never been different in the other experiments. Given is the mean ± S.E.M, and, in italics, the *p*-values of the Mann–Whitney rank sum test. For pyramidal cells in **(A)**: ADL, apical dendritic length [μm]; BDL, the average basal dendritic length per cell [μm]; the number of dendritic segments; for interneurons in **(B)**: MDL, mean dendritic length [μm] per cell; MDS, mean dendritic segments; no. of PD, number of primary dendrites; *n*, number of neurons analyzed.*

**TABLE 5:** Ifenprodil treatment decreased GAD expression and increased phosphorylation of GluN2B.

**Protein**	**Control**	**Ifenprodil**	**12 h recovery**	**24 h recovery**	***p***
GluN2B	1.0 ± 0.06 (6)	1.05 ± 0.05 (3)	n.d.	1.04 ± 0.06 (3)	*n.s.*
ph-GluN2B	1.0 ± 0.04 (10)	n.d.	**1.23 ± 0.052** (5)	**1.26 ± 0.049** (6)	*0.009*
GluN1	1.0 ± 0.02 (3)	n.d.	1.04 ± 0.01 (3)	1.02 ± 0.02 (3)	*n.s.*
PSD-95	1.0 ± 0.038 (19)	1.09 ± 0.041 (6)	1.034 ± 0.06 (6)	0.98 ± 0.019 (11)	*n.s.*
GAD-67	1.0 ± 0.027 (18)	**0.88 ± 0.02** (6)	**0.89 ± 0.014** (7)	0.99 ± 0.016 (12)	*<0.001*
GAD-65	1.0 ± 0.016 (18)	**0.78 ± 0.038** (6)	**0.84 ± 0.015** (7)	0.92 ± 0.013 (12)	*<0.001*

*OTC from three preparations were either treated with ifenprodil [10 μM] from DIV 6–10, and harvested at DIV 10 (ifenprodil), or washed two times with fresh medium and maintained for 12 and 24 h (recovery) without ifenprodil. Control cultures were mock-stimulated with water. For the recovery, control cultures were harvested at the 24 h time point. Gel by gel, to generate a S.E.M. also for the controls, control values were normalized to the average of each group of control values which was set to 1; thereafter, all controls were pooled. Reported are the mean ± S.E.M. The number of blots run for every protein/epitope is given in brackets. Statistical comparison was done by ANOVA on ranks versus control; in italics, *p-*values are given. In bold, values significantly different from control; n.s., not significant; n.d., not determined.*

### Attempts to Overexpress NMDAR Subunits Were Unsuccessful in Rat Cortical OTC

**FIGURE 7:**
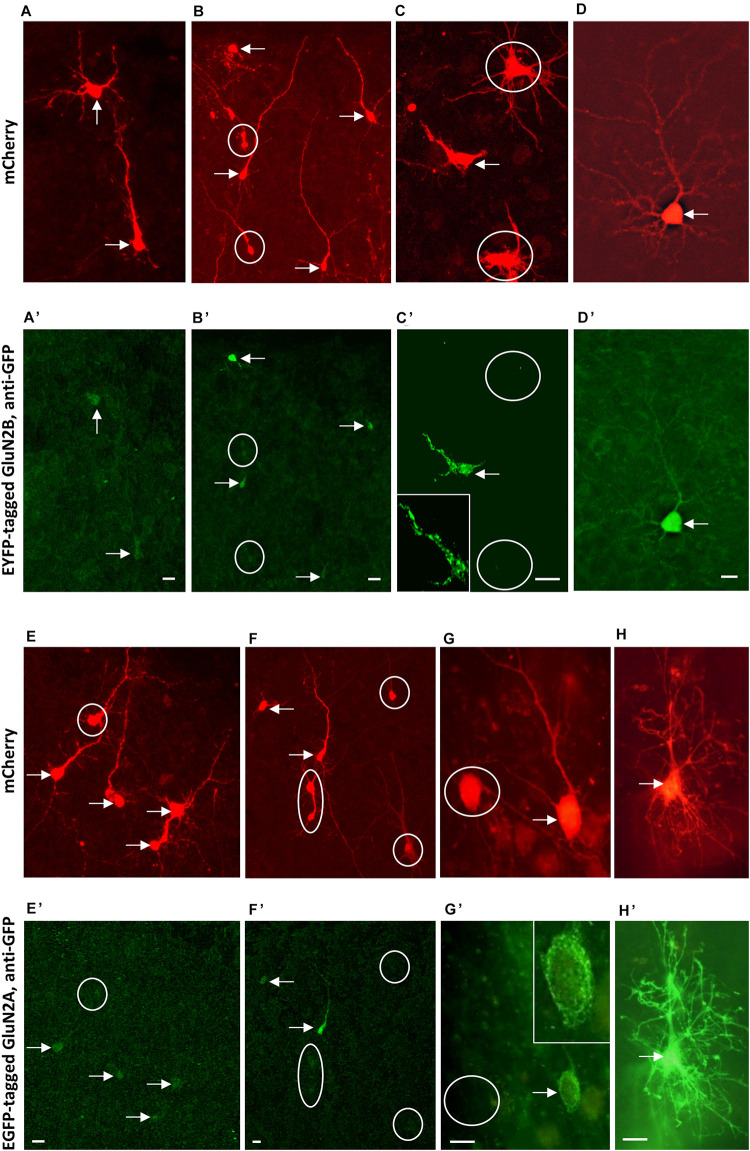
Immunofluorescence staining of overexpressed tagged GluN2 subunits. **(A–D)** Co-transfection of mCherry (mCherry fluorescence) **(A–D)** and EYFP-tagged GluN2B-encoding plasmids stained with mouse anti-GFP/Alexa-488 **(A’–D’)**. **(E–H)**: Co-transfection of mCherry (mCherry fluorescence) **(E–H)** and EGFP-tagged GluN2A-encoding plasmids stained with mouse anti-GFP/Alexa-488 **(E’**–**H’)**. Note intense mCherry staining of immature as well as more mature neurons and the barely visible GluN2A and GluN2B signal. Intensity of the GluN2A and GluN2B immunofluorescence staining increases in the pictures arranged from left to right, and the right-most cells [the pyramidal neuron in **(D,D’)**, and the astrocyte in **(H,H’)**] had in fact been the two cells we found most intensely labeled for the subunits. Note that the tag fluorescence appeared rather cytosolic, also occluding the nucleus. Double-labeled cells marked by arrows, mCherry-positive cells without GluN2 fluorescence marked by white circles. About 10 OTC transfected from two preparations for each GluN2 subunit; sequential confocal imaging. Pial surface is to the top for all pictures. Scale: 10 μm.

Following the strategy successfully employed for AMPAR and KAR subunits (Hamad et al., 2011; Jack et al., 2018) we attempted to overexpress GluN2B and GluN2A subunits from DIV 5–10. An elevated expression of GluN2B during development and through adulthood results in a smarter mouse (Tang et al., 1999). We hypothesized an increase in basal dendritic complexity. Unexpectedly, pyramidal neurons and multipolar interneurons transfected with either GluN2A- or GluN2B-encoding plasmids resulted in dendritic dimensions that were not different from those of EGFP-only control neurons (Table 6). Not even trends e.g., toward longer or more complex branches were seen. We questioned if the subunits were produced by the neurons. We transfected EGFP-tagged GluN2A and EYFP-tagged GluN2B into neurons in OTC, however, the tag fluorescence was barely detectable in some few transfectants. Only immunostaining with anti-EGFP antibody delivered a rather faint fluorescence in cell bodies and proximal processes of a minority of the often brilliantly mCherry-positive transfected neurons and glia cells (Figures 7A–H). Labeling appeared cytosolic and plasma membrane-associated, occluded the nucleus, and sometimes revealed intracellular aggregates (Figures 7C,C’,G,G’) which might suggest retention of the tagged proteins at the endoplasmatic reticulum.

**TABLE 6:** Effects of transfection with plasmids encoding GluN2A and GluN2B.

**(A) Pyramidal cells transfected with GluN2A and GluN2B encoding plasmids from DIV 5–10**				
	**Pyramidal cells of layers II/III**		**Pyramidal cells of layers V/VI**	
**Condition (no. of batches)**	**ADL (*n*) Segments**	**BDL Segments**	**ADL (n) Segments**	**BDL Segments**
Control	1248 ± 52 (93) 29 ± 1.4	213 ± 10 5.6 ± 0.3	1024 ± 38 (118) 19 ± 0.7	261 ± 13 5.3 ± 0.2
GluN2A (4)	1132 ± 46 (65) 26 ± 1.3	256 ± 15 6.1 ± 0.4	1065 ± 39 (81) 19 ± 0.8	286 ± 15 5.6 ± 0.3
GluN2B (4)	1183 ± 47 (100) 27 ± 1.2	253 ± 17 6.0 ± 0.4	1051 ± 30 (114) 20 ± 0.7	265 ± 12 5.9 ± 0.3
*ANOVA on ranks vs. control*	*P = 0.58 P = 0.47*	*P = 0.09 P = 0.51*	*P = 0.3 P = 0.21*	*P = 0.33 P = 0.10*

**(B) Interneurons transfected with GluN2A and GluN2B encoding plasmids from DIV 5–10**				
**Condition (no. of batches)**	**MDL (n)**	**MDS**		**no. of PD**

Control	362 ± 16 (81)	6.1 ± 0.3		5.1 ± 0.2
GluN2A (4)	375 ± 17 (90)	6.2 ± 0.3		4.8 ± 0.2
GluN2B (4)	409 ± 21 (73)	6.9 ± 0.3		5.0 ± 0.2
*ANOVA on ranks vs. control*	*P = 0.32*	*P = 0.06*		*P = 0.61*

*Given is the mean ± S.E.M, and, in italics, the *p*-values of an ANOVA on ranks. For pyramidal cells in **(A)**: ADL, apical dendritic length [μm]; BDL, the average basal dendritic length per cell [μm]; the number of dendritic segments; for interneurons in **(B)**: MDL, mean dendritic length [μm] per cell; MDS, mean dendritic segments; no. of PD, number of primary dendrites; *n*, number of neurons analyzed.*

The overexpression of AMPAR and KAR subunits confers an elevated sensitivity toward the ligands with dendritic beading as quantitative readout (Hamad et al., 2011; Jack et al., 2018). Thus, cultures transfected with GluN2A and GluN2B, respectively, from DIV 5–10 were challenged with 50 μM NMDA for 10 min. Counting revealed that dendritic injury in pyramidal neurons and multipolar non-pyramidal neurons occurred to similar degrees that were statistically not different from the proportion of injured cells evoked in EGFP-only cultures [mean + S.E.M in (*n*) OTC; for pyramidal cells, control: 63.2 + 3.8% (11), GluN2A: 58.4 + 8.5% (9), GluN2B: 64.0 + 7.8% (9); ANOVA on ranks *p* = 0.916; for interneurons, control: 51.3 + 13.6%, GluN2A: 45.7 + 10.5%, GluN2B: 53.1 + 8.1%; ANOVA on ranks *p* = 0.21].

To confirm that the tagged NMDA receptor channels are functional we transfected HEK cells for immunostaining and patch-clamp recordings. At 24–48 h post-transfection (together with GluN1 which is not endogenously present in HEK cells as compared to neurons) the EYFP-GluN2B and the EGFP-GluN2A tag fluorescence was barely detectable in HEK cells. As in neurons, only the immunofluorescence for the tag with anti-GFP antibodies yielded a weak to moderate staining of HEK cells along the membrane and sometimes clustered within the cytosol (Supplementary Figures 1A,B). Albeit small, currents were present. Application of 500 μM glutamate elicited typical inward currents with the inactivation being less pronounced in GluN1/GluN2B-transfected HEK cells (Supplementary Figures 2A–C). Peak currents were similar and steady-state currents were larger in GluN1/GluN2B-transfected HEK cells (Supplementary Figures 2D,E). This confirmed that the NMDA receptor constructs encode functional channels.

Finally, to test if the endogenous GluN1 expression could be a limiting factor in neurons, we co-transfected GluN1-1a together with GluN2B encoding plasmids from DIV 5-10. GluN1-1a is abundantly expressed postnatally (Laurie and Seeburg, 1994). To this end, focusing on the reconstruction of supragranular pyramidal neurons, neither GluN1-1a alone nor GluN1-1a/GluN2B expression resulted in an altered dendritic complexity (Table 7).

**TABLE 7:** Effects of transfection with plasmids encoding GluN1-1a and GluN1-1a/GluN2B from DIV 5–10.

**Effects of overexpression of GluN1-1A and GluN1-1A/GluN2B**		
	**Pyramidal cells of layers II/III**	
**Condition (no. of batches)**	**ADL (*n*) Segments**	**BDL Segments**
Control	1045 ± 78 (37) 26 ± 1.5	243 ± 17 6.7 ± 0.4
GluN1-1A (2)	1064 ± 61 (27) 25 ± 1.6	280 ± 22 7.6 ± 0.3
GluN1-1A/GluN2B (2)	1147 ± 57 (42) 26 ± 1.7	237 ± 16 6.5 ± 0.5
*ANOVA on ranks vs. control*	*P = 0.24 P = 0.95*	*P = 0.22 P = 0.27*

*Pyramidal cells of layers II/III have been assessed. Given is the mean ± S.E.M, and, in italics, the *p*-values of an ANOVA on ranks. ADL, apical dendritic length [μm]; BDL, the average basal dendritic length per cell [μm]; the number of dendritic segments; *n*, number of neurons analyzed.*

Taken together, the fairly weak presence of the transfected GluN2 proteins in HEK cells and in neurons, no enhanced sensitivity of the neuronal transfectants to NMDA, and their unchanged dendritic complexity suggested that GluN2 transfections did not result in a substantially increased production of functional GluN2 proteins in spontaneously active postnatal neocortical neurons.

## Discussion

The present study reports first, a class-specific effect on dendritic morphology in that only pyramidal cells are affected, second, a compartment effect with basal dendrites being affected, and third, an effect mediated by GluN2B-containing receptors that is limited to an early time window. The importance of neural activity and glutamate receptors for neuron morphology has been demonstrated in cortex and hippocampus of mammals as well as the frog optic tectum (Cline, 2001). Yet, the question of which subunits may or may not elicit certain effects, in which dendritic compartments they occur and in what developmental time windows has not been fully resolved. For instance, after blocking transmitter release by tetanus toxin injections into newborn rat hippocampus stratum oriens and stratum radiatum, CA1 pyramidal cells display less branched basal, but normal apical dendrites at day 6–10 (Groc et al., 2002). Ablation of NMDARs impairs dendritic maturation by altering the membrane excitability, a critical component for dendrite growth (Frangeul et al., 2017). Hippocampal granule cells with fragile-X-associated NMDAR hypofunction display reduced dendritic length and branching (Yau et al., 2019). Blocking NMDARs from P1-P21 by oral supplementation of the non-selective antagonist CGP 40116 results in stunted basal dendrites of adult prefrontal cortical pyramidal cells suggesting an inability to recover with time; apical dendrites had been either not altered or have managed to recover a normal morphology (Wedzony et al., 2005).

Antagonizing GluN2-containing receptors between DIV 5–10 impaired basal dendrites by slowing the growth and/or freezing the dendrites to the degree of complexity they had reached at the start of the treatment. Yet, growth resumes after ifenprodil was removed, and quickly catches up to the age-matched control level. Blots suggested that it was possibly mediated by the transient deficit of GABA-ergic inhibition as suggested by the reduction and delayed recovery of GAD-65/67 expression and by a higher synaptic GluN2B signaling as suggested by the enhanced Y1472 phosphorylation. In the presence of ifenprodil the neurons might have increased the density of synaptic GluN2B-containing receptors by homeostatic mechanisms. Synaptic currents are particularly important for dendritic growth and remodeling (Wong and Ghosh, 2002).

The specific effect on basal dendrites was surprising. GluN2A and GluN2B receptors are fairly equally distributed in apical and basal dendrites, as has been shown for layer V prefrontal cortical pyramidal cells, albeit of postnatal day 21–33 mice (Balsara et al., 2014) which are older than our neurons. Further, blocking NMDARs with APV has been shown to neutralize the growth-promoting effect delivered by certain AMPAR subunits on the apical dendrites (Hamad et al., 2011), but APV did not block basal growth in this experimental setting. Biophysical differences exist between basal and apical dendrites (Schiller et al., 2000; Milojkovic et al., 2005; Nevian et al., 2007; Major et al., 2008; Antic et al., 2010). For instance, basal dendrites receive a major fraction of intracortical excitatory synapses, NMDA spikes occur somewhat enriched in basal dendrites (Chalifoux and Carter, 2011) and evoke large calcium transients (Schiller et al., 2000) which are linearly related to the neuron’s spike activity (Hill et al., 2013). Further, excited basal dendrites have been found to elicit sustained UP states in the neuron’s cell body (Milojkovic et al., 2005; Antic et al., 2010). UP states of higher excitability are regarded as important triggers of transcriptional and translational processes required for dendritic growth. Yet, a balanced NMDAR signaling seems to be important. In hippocampal slice cultures, a 4-days chronic disinhibition with bicuculline at an age equivalent to our DIV 10 cortex cultures impairs elongation and branching of basal dendrites. This can be prevented by APV suggesting that hyperexcitability with impairment of CREB signaling (Nishimura et al., 2008) is as detrimental as hypoexcitability under GluN2B inhibition.

The temporal limitation was presumably caused by the time course of subunit expression. The developmental decrease of GluN2B protein is relative (Williams et al., 1993; Traynelis et al., 2010; Sanz-Clemente et al., 2013), and in fact the assembly of GluN1/GluN2B raises quite steadily until P20 in rat cortex as determined by immunoprecipitation (Babb et al., 2005) with surface expression of GluN2B being independent of activity. GluN2A protein increases strongly during the first postnatal week, and this proceeds also in OTC (Neumann et al., 2019). Given the presence of spontaneous activity, GluN2A efficiently replaces GluN2B in GluN2B-containing receptors at the cell surface (Barth and Malenka, 2001; Barria and Malinow, 2002). Yet, a majority of synaptic NMDAR in cortex and hippocampus seem to be GluN1/2A/2B triheteromeric receptors (Stroebel et al., 2018). Extrasynaptic NMDARs also contain GluN2B (Papouin and Oliet, 2014). Early in development dendrites have very few spines, and receptors are not necessarily clustered. What in adult neurons is an extrasynaptic NMDA receptor may in a developing neuron be a receptor at a site where a post synapse is going to form or has just been eliminated (Petralia et al., 2010). Along that line, we could trigger massive dendritic injury in pyramidal neurons in the early time window. Extrasynaptic GluN2B receptors contribute to excitotoxicity (Hardingham and Bading, 2010), but also to calcium plateau potentials (Oikonomou et al., 2012) which can influence early dendritic growth dynamics (Engert and Bonhoeffer, 1999; Maletic-Savatic et al., 1999; Kwon and Sabatini, 2011).

Growth requirements of GABA-ergic interneurons have rarely been analyzed. Of nine AMPAR subunits tested, exclusively the GluA1(Q)-flip subunit has been able to increase dendritic complexity of interneurons (Hamad et al., 2011). Further, interneuronal dendritic growth is promoted by GluK1 (Jack et al., 2018). Fast-spiking as well as regular-spiking non-pyramidal neurons express NMDAR (Hadzic et al., 2017) and undergo the subunit switch (Wang and Gao, 2009). An impairment of interneuronal dendrites has been reported in mutant Dlx1/2 mice with evidence that a reduction of GluN2B expression is the cause (Pla et al., 2018). This is in contrast to our findings that neither the block of GluN2B nor of GluN2A could alter interneuronal dendritic complexity. Opposed to GluN1/2A/2B-containing pyramidal cells, interneurons are more enriched for GluN1/2B/2D-containing receptors, and the efficacy of ifenprodil at GluN1/2B/2D receptors is much less than at GluN1/2B receptors (Yi et al., 2019). A recent study in mouse cortex has revealed that parvalbuminergic interneurons mature via tonically active GluN2C/2D-containing receptors which transform the depolarizing signaling of ambient glutamate into an increase of dendritic complexity. Interestingly, the action is temporally limited to an early postnatal time window: a pharmacological block of GluN2C/2D receptors between day 7 and 9 yields at day 21 interneurons with less dendritic branch points and reduced inhibitory activity (Hanson et al., 2019).

The attempt to overexpress GluN2B and GluN2A subunits via CMV-driven plasmids in spontaneously active cortical slice cultures did not alter dendritic geometry, not even after co-transfection with GluN1-1a. The transfectants were still present and look healthy suggesting that there was no cell death upon overexpression. Yet, staining for the tagged subunits gave only weak immunosignals of the transfectants, HEK cells and neurons. Further, although the HEK cell recordings demonstrated that the NMDA receptor constructs encode functional channels, the neuronal transfectants had no higher sensitivity to NMDA. In contrast, AMPAR subunits and TARPs overexpressed by the same strategy from plasmids with the same backbone resulted in well stained neurons, membrane labeling, and a higher sensitivity to AMPA (Hamad et al., 2011, 2014).

Results of studies employing GluN2 subunit overexpression are controversial. Transgenic GluN2B overexpressing mice display enhanced Ro25-6981 dependent LTP in frontal cortex (Cui et al., 2011), but visual cortical neurons of GluN2B-overexpressing mice display parameters of synaptic plasticity not any different from wildtype control (Philpot et al., 2001), suggestive of areal differences. Toward morphofunctional differentiation, reported effects seem to depend on timing and endogenous receptor status. Ventral spinal cord neurons dissociated from E14 rat embryos downregulate NMDARs, and undergo an enhanced dendritic growth upon transfection with GluN2B, but not GluN2A (Sepulveda et al., 2010). Hippocampal neurons richly express GluN2 receptors, and here, the knockdown of GluN2B results in a slight decrease in number of only the secondary dendrites whereas knockdown of GluN2A increases the number of such secondary branches (Sepulveda et al., 2010). In the tadpole tectum, the knockdown of GluN2B has no effect on dendritic development (Ewald et al., 2008). Overexpressing GluN2B promotes branching in dissociated hippocampal neurons at DIV 7, but no longer in older neurons (Bustos et al., 2014). A GluN2B knockdown reduces dendritic complexity (Bustos et al., 2017). Genetic ablation of GluN2B does not affect dendritic dimensions of dentate granule cells and barrel cortex layer IV spiny stellates, but the lack of GluN2B prevents the reorganization of spiny stellate dendrites toward the preferred barrel (Espinosa et al., 2009). Moreover, the effects of overexpression are sometimes surprisingly small. For instance, in tadpole tectum neurons overexpressing GluN2 receptors, recordings reveal a shift to either one of the two subunits, but total branch length and branch tip numbers remain at control level, and only a small difference of the distance between dendritic end points has been found (Ewald et al., 2008). The biological significance of such a difference remains up to debate. The amount of GluN2B at the surface is difficult to change since synaptic incorporation can neither be increased by enhancing subunit expression (Philpot et al., 2001; Barria and Malinow, 2002), nor by blocking AMPARs (Von Engelhardt et al., 2009). Our results now add to this showing that the total amount of GluN2B and GluN1 protein were not altered under ifenprodil and during recovery from ifenprodil treatment. Possibly, the overexpression-evoked surface levels of GluN2 proteins are large enough to be detectable with sensitive recordings, and this would be supported by our HEK cell recordings. However, in neurons, such currents presumably are not large enough to measurably alter the dendritic architecture. Another reason could be that we transfected wildtype GluN2B. Possibly, only the overexpression of an endocytosis-resistant E1479Q mutant (De Solis et al., 2019) might result in a GluN2B surface expression large enough to evoke morphological effects. Other mental disorder-related mutants of GluN2B have been recently discovered. For instance, GluN2B truncated at position 724 assembles with GluN1 but fails to reach the plasma membrane, and overexpressing this mutant in dissociated rat cortical pyramidal neurons results at DIV 10–14 in dramatically stunted dendritic growth (Sceniak et al., 2019). Further, a recent study of transgenic mice expressing chimeric GluN2 subunits reports that overexpression of the GluN2A with the GluN2B C-terminus, but not N-terminus or transmembrane domains, results in longer dendrites of CA1 pyramidal neurons suggesting that particular aspects of intracellular, i.e., metabotropic signaling rather than the ion conductance *per se* causes these effects (Keith et al., 2019). For instance, nanomolar concentrations of kainate drives dendritic growth via a metabotropic action (Jack et al., 2018), and changes of spine morphology are evoked by non-ionotropic actions of NMDARs because the changes persist in the presence of antagonists (Stein et al., 2015, 2020). The role of mutant GluN2B or non-ionotropic actions for postnatal maturation of basal dendrites remains to be unraveled. To this end, it appears as if young neocortical neurons in spontaneously active slice cultures strictly regulate the amount of functional NMDARs, possibly as a trade-off between growth and toxicity.

## Data Availability Statement

The original contributions presented in the study are included in the article/Supplementary Material, further inquiries can be directed to the corresponding author.

## Ethics Statement

All animal protocols were approved by the Ruhr University Bochum Animal Research Board and the State of North Rhine-Westphalia.

## Author Contributions

MIKH, MH, ARe, and PW designed the experiments. ARä and SK prepared the cultures and solutions. SG, JG, AS, FW, RB, OK, TZ, CR, MIKH, and PW performed the experiments, reconstructions, and data management. SG, JG, MIKH, ARe, and PW interpreted the results. PW wrote the manuscript. SG, JG, OK, MIKH, ARe, and MH contributed to writing and gave feedback on the manuscript. All authors approved the final version.

## Conflict of Interest

The authors declare that the research was conducted in the absence of any commercial or financial relationships that could be construed as a potential conflict of interest.
