# Supplementary material for: GluN2B but Not GluN2A for Basal Dendritic Growth of Cortical Pyramidal Neurons
Source: Front Neuroanat. 2020 Nov 13;14:571351. doi: 10.3389/fnana.2020.571351 (PMC7691608; doi:10.3389/fnana.2020.571351)

**Supplemental Table 1. Effects of the ampakine CX546 and Ifenprodil on pyramidal cells of layers II/III at DIV 10.** Given is the mean ± S.E.M, and, in italics, the p-values of an ANOVA on ranks. For pyramidal cells in **(A)**: ADL, apical dendritic length [µm]; BDL, the average basal dendritic length per cell [µm]; the number of dendritic segments; for interneurons in **(B)**: MDL, mean dendritic length [µm] per cell; MDS, mean dendritic segments; no. of PD, number of primary dendrites; n, number of neurons analyzed. In bold, the parameters that differ significantly from control.

| **Supplemental Table 1**  **A. Pyramidal cells of layers II/III treated with CX546 and ifenprodil from DIV 5-10** | | | |
| --- | --- | --- | --- |
| Condition  (no. of batches) | ADL ( n )  Segments | BDL  Segments | |
| Control | 1089 ± 70 ( 23 )  23 ± 1.4 | 265 ± 25  6.8 ± 0.5 | |
| CX546 (2) | 1153± 60 ( 27 )  23 ± 1.8 | 295 ± 30  6.3 ± 0.5 | |
| CX546 + ifenprodil (2) | 1022 ± 78 ( 26 )  23.2 ± 2.4 | **186 ± 27**  **5.1 ± 0.7** | |
| *ANOVA on ranks vs control* | *P = 0.29*  *P = 0.82* | *P = 0.004*  *P = 0.038* | |
| **B. Interneurons treated with CX546 and ifenprodil from DIV 5-10** | | | |
| Condition  (no. of batches) | MDL ( n ) | MDS | no. of PD |
| Control | 389 ± 40 ( 18 ) | 8.3 ± 0.8 | 4.7 ± 0.2 |
| CX546 (2) | 367 ± 30 ( 21 ) | 7.6 ± 0.5 | 4.5 ± 0.3 |
| CX546 + ifenprodil (2) | 341 ± 23 ( 32 ) | 7.3 ± 0.8 | 5.0 ± 0.3 |
| *ANOVA on ranks vs control* | *P = 0.5* | *P = 0.13* | *P = 0.5* |

**Supplemental Table 2. Effects of the ampakine CX546 at DIV 20.** Given is the mean ± S.E.M, and, in italics, the p-values of a Mann-Whitney rank sum test. For pyramidal cell in **(A)**: ADL, apical dendritic length [µm]; BDL, the average basal dendritic length per cell [µm]; the number of dendritic segments; and for interneurons in **(B)**: MDL, mean dendritic length [µm] per cell; MDS, mean dendritic segments; no. of PD, number of primary dendrites; n, number of neurons analyzed.

| **Supplemental Table 2**  **A. Pyramidal cells treated with CX546 from DIV 10-20** | | | | | | |
| --- | --- | --- | --- | --- | --- | --- |
|  | Pyramidal cells in layers II/III | | | Pyramidal cells in layers V/VI | | |
| Condition  (no. of batches) | ADL ( n )  Segments | BDL  Segments | | ADL ( n )  Segments | | BDL  Segments |
| Control | 1412 ± 102 ( 28 )  30 ± 2.4 | 302 ± 10  7.2 ± 0.4 | | 1332 ± 67 ( 33 )  23 ± 1.5 | | 326 ± 17  6.7 ± 0.5 |
| CX546 (2) | 1325 ± 96 ( 23 )  30 ± 2.6 | 341 ± 25  8.1 ± 0.6 | | 1353 ± 77 ( 34 )  26 ± 1.5 | | 385 ± 29  8.2 ± 0.6 |
| *Mann-Whitney test vs control* | *P = 0.84*  *P = 0.85* | *P = 0.31*  *P = 0.29* | | *P = 1.00*  *P = 0.14* | | *P = 0.19*  *P = 0.1* |
| **B. Interneurons treated with CX546 from DIV 10-20** | | | | | | |
| Condition  (no. of batches) | MDL ( n ) | | MDS | | no. of PD |  |
| Control | 538 ± 35 ( 33 ) | | 6.6 ± 0.4 | | 3.9 ± 0.2 |  |
| CX546 (2) | 476 ± 40 ( 24 ) | | 5.8 ± 0.4 | | 3.7 ± 0.2 |  |
| *Mann-Whitney test vs control* | *P = 0.26* | | *P = 0.3* | | *P = 0.64* |  |

**Supplemental Table 3. Individual effects of the four antagonists on pyramidal neurons at DIV 10.** Data presented separately to show that the two GluN2B antagonists elicited comparable effects. Cumulative controls (from Table 2) are given for comparison. Given is the mean ± S.E.M. ADL, apical dendritic length [µm]; BDL, the average basal dendritic length per cell [µm]; the number of dendritic segments; n, number of cells analyzed.

| **Supplemental Table 3**  **Pyramidal cells treated with ifenprodil, Ro25-6981, TCN201 and NVP-AAM077 from DIV 7-10** | | | | |
| --- | --- | --- | --- | --- |
|  | Pyramidal cells of layers II/III | | Pyramidal cells of layers V/VI | |
| Condition  (no. of batches) | ADL ( n )  Segments | BDL  Segments | ADL ( n )  Segments | BDL  Segments |
| Control | 1167 ± 50 ( 89 )  32 ± 1.5 | 244 ± 13  7.0 ± 0.4 | 1016 ± 37 ( 75 )  23 ± 1.2 | 272 ± 14  6.8 ± 0.3 |
| Ro25-6981 (3) | 1040 ± 77 ( 38 )  30 ± 2.1 | 224 ± 12  6.6 ± 0.4 | 992 ± 67 ( 40 )  22 ± 1.3 | 208 ± 14  5.7 ± 0.4 |
| Ifenprodil (3) | 1041 ± 41 ( 49 )  25 ± 1.3 | 173 ± 10  5.3 ± 0.35 | 1008 ± 52 ( 37 )  22 ± 1.9 | 203 ± 14  5.3 ± 0.35 |
| Control | 1193 ± 48 ( 101 )  32 ± 1.3 | 237 ± 11  6.9 ± 0.3 | 1014 ± 39 ( 64 )  22 ± 1.2 | 269 ± 14  7.1 ± 0.4 |
| NVP-AAM077 (3) | 1115 ± 47 ( 60 )  31 ± 1.5 | 236 ± 11  7.0 ± 0.33 | 1118 ± 49 ( 49 )  25 ± 1.4 | 267 ± 17  7.0 ± 0.43 |
| TCN201 (2) | 1134 ± 77 ( 25 )  32 ± 2.7 | 209 ± 17  6.9 ± 0.58 | 886 ± 65 ( 18 )  22 ± 1.7 | 215 ± 18  5.2 ± 0.5 |

**Supplemental Figure 1. Immunocytochemical detection of NMDA receptor constructs in HEK293T cells.** **(A)** Immunocytochemical staining of HEK cells transfected with GluN1 and GluN2A-GFP subunits and **(B)** transfected with GluN1 and GluN2B-GFP subunits. (*Left*) GFP fluorescence was not or only hardly detectable. (*Middle - right*) The signal obtained after staining with anti-GFP antibody followed by Alexa Fluor 568-labeled secondary antibody indicates NMDA receptor expression in several cells. The overlay with the DAPI channel (rightmost pictures) shows that the localization of the red anti-GFP signal is consistent with GluN2A-GFP localization at the cell membrane, whereas most of the GluN2B immunoreactive material seemed to accumulate intracellularly.

**
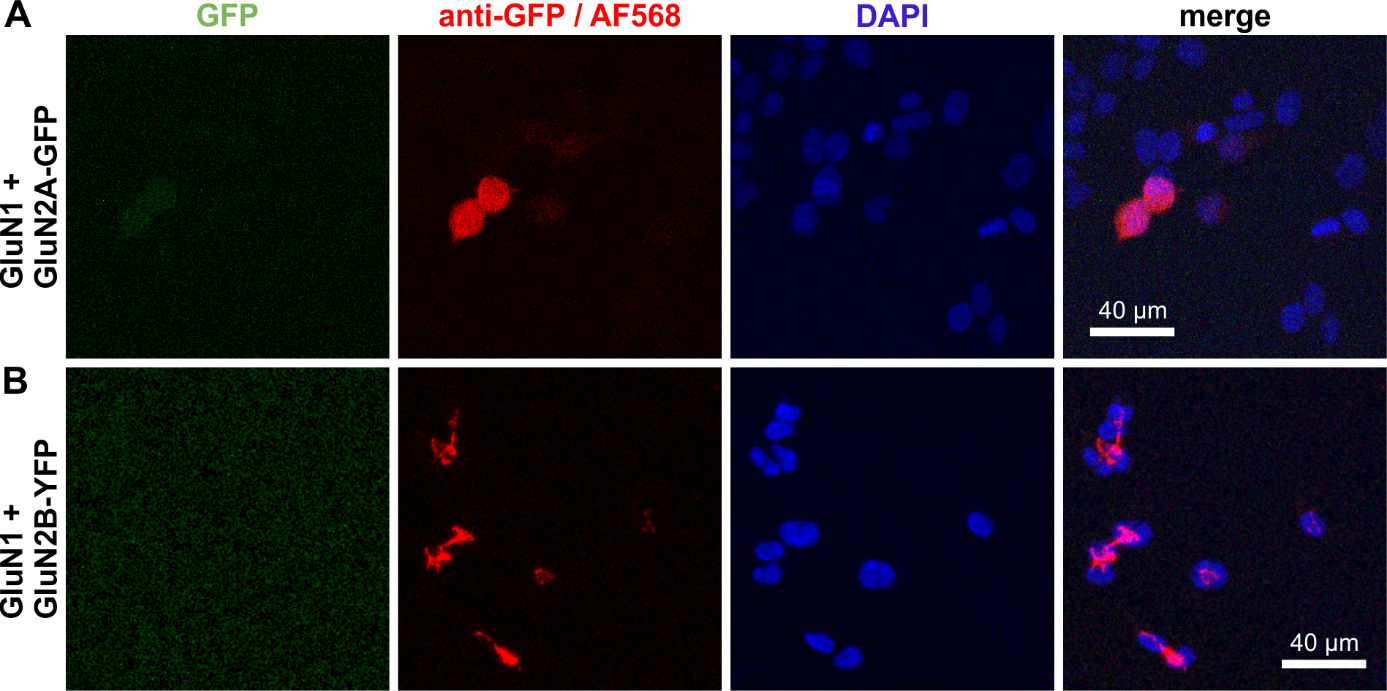
**

**Supplemental Figure 2. Functional expression of NMDA receptor constructs in HEK293T cells.** Whole-cell voltage clamp recordings were used to assess the functional expression of the GluN2A and GluN2B constructs in HEK cells. **(A)**  Application of 500 μM glutamate to a cell co‑expressing GluN1/GluN2A subunits results in typical inward currents. The current shows some inactivation and an increased noise level due to the large single-channel conductance of NMDA receptors. **(B)**  Application of 500 μM glutamate to a cell co-expressing GluN1/GluN2B subunits. As expected, the inactivation is less pronounced than in GluN1/GluN2A receptors. **(C)**  Control application of 500 μM glutamate to an untransfected HEK cell. **(D,E)**  Quantification of current responses and steady-state currents. The bars indicate the mean peak currents (GluN2A 126 ± 90 pA; GluN2B 105 ± 74 pA; mean ± S.D.) and mean steady-state currents (GluN2A 32 ± 7 %; GluN2B 71 ± 15 %; mean ± S.D.). The number of analyzed cells is given in parentheses. The holding potential was ‑70 mV and 50 µM glycine was present throughout the recordings.


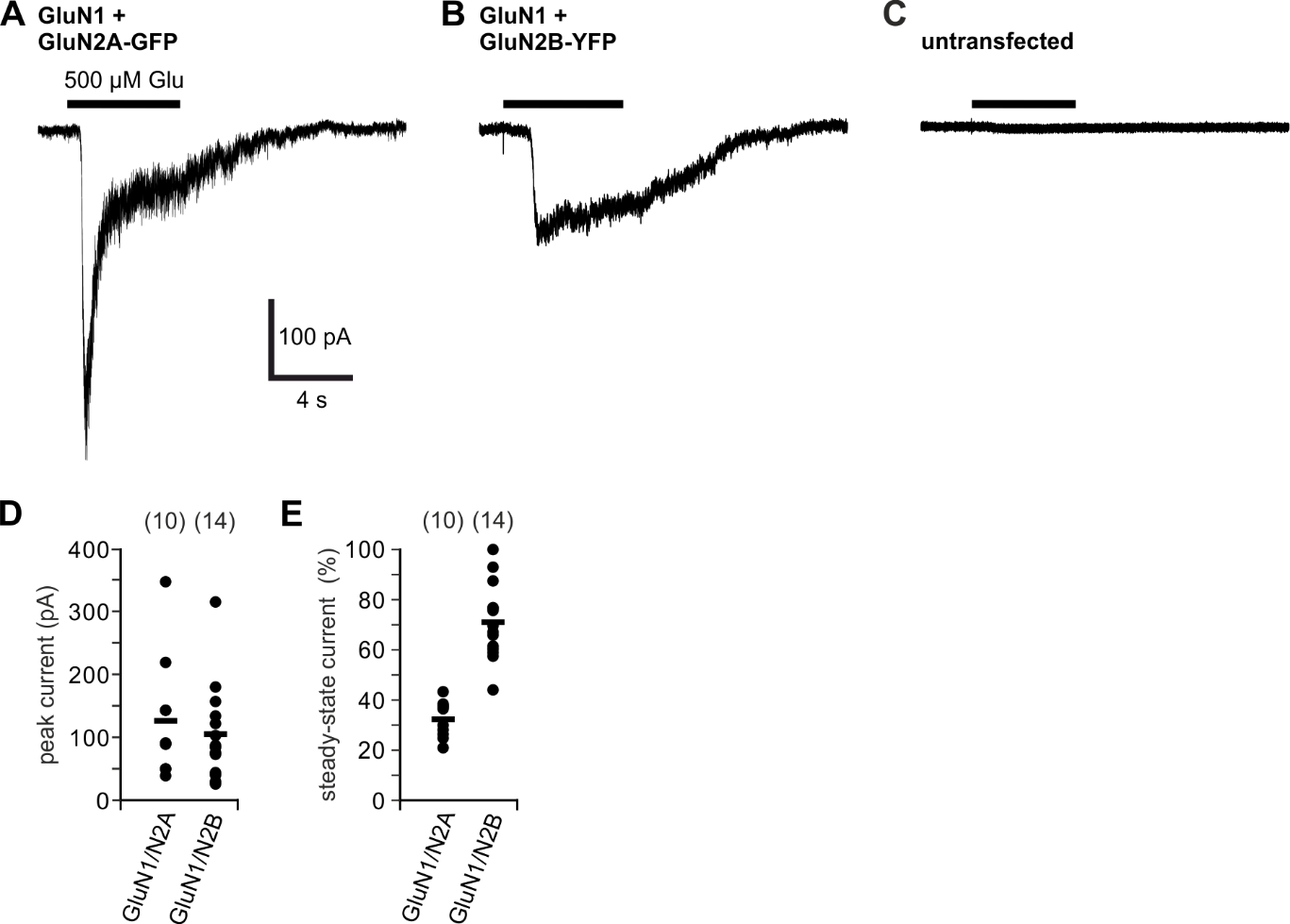

Supplement: Supplementary file 1 [file Data_Sheet_1.docx]
